# Supplementary material for: Efficient Selection of Antibodies Reactive to Homologous Epitopes on Human and Mouse Hepatocyte Growth Factors by Next-Generation Sequencing-Based Analysis of the B Cell Repertoire
Source: Int J Mol Sci. 2019 Jan 18;20(2):417. doi: 10.3390/ijms20020417 (PMC6359367; doi:10.3390/ijms20020417)

Supplementary figure 1

Figure S1. Component statistics. (a) Rank abundance of vertices in each dataset. **(b)** Rank clonal frequency of components in each dataset. The clonal frequency of each vertex indicates the read count in each vertex divided by the total read count in the dataset containing the vertex.

a

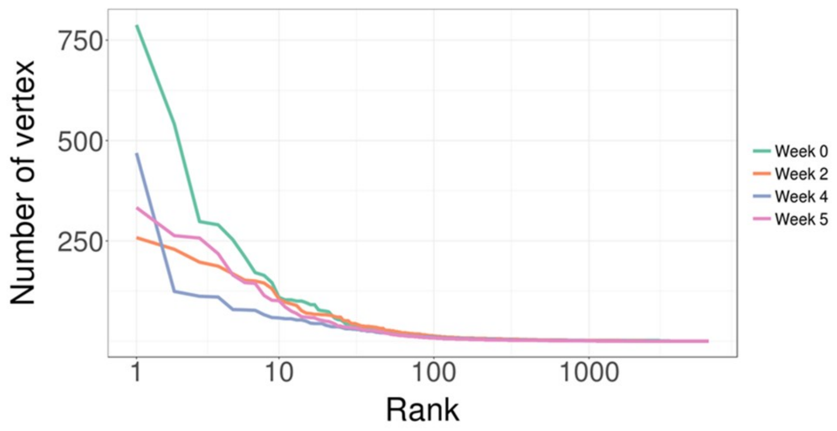

750
500
250
Number of vertex
1
10
100
1000
Rank
Week 0
Week 2
Week 4
Week 5

b

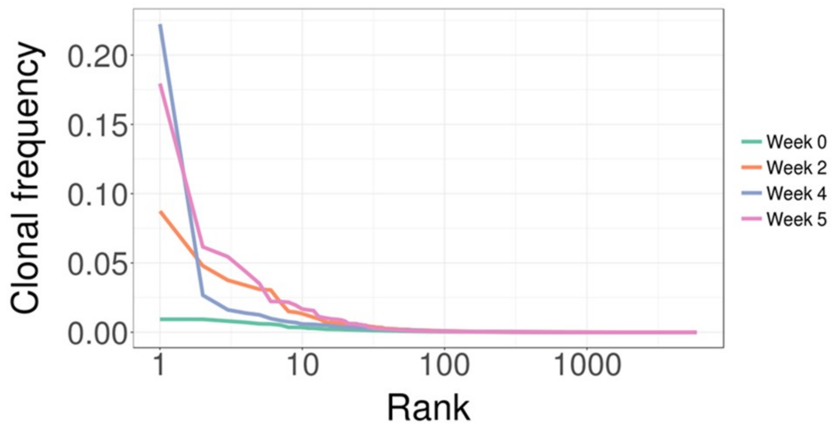

0.20
0.15
0.10
0.05
0.00
Clonal frequency
1
10
100
1000
Rank
Week 0
Week 2
Week 4
Week 5

Supplementary figure 2

Figure S2**. Network image of V_H components at weeks 0, 2, 4, and 5 in high resolution**

Network image of V_H components at week 0 **(a)**, 2 **(b)**, 4 **(c)**, and 5 **(d). (e)** Network image of total V_H components in all f our sets. In all network image, the vertices were connected b y finding minimum spanning trees. Components composed of a single vertex were excluded. Vertices and edges of com ponents showing significant increase in number of vertex an d/or clonal frequency on the scatter plot are labeled in brow n. Components are located in the same position throughout the network images.

Supplementary Figure 2
a

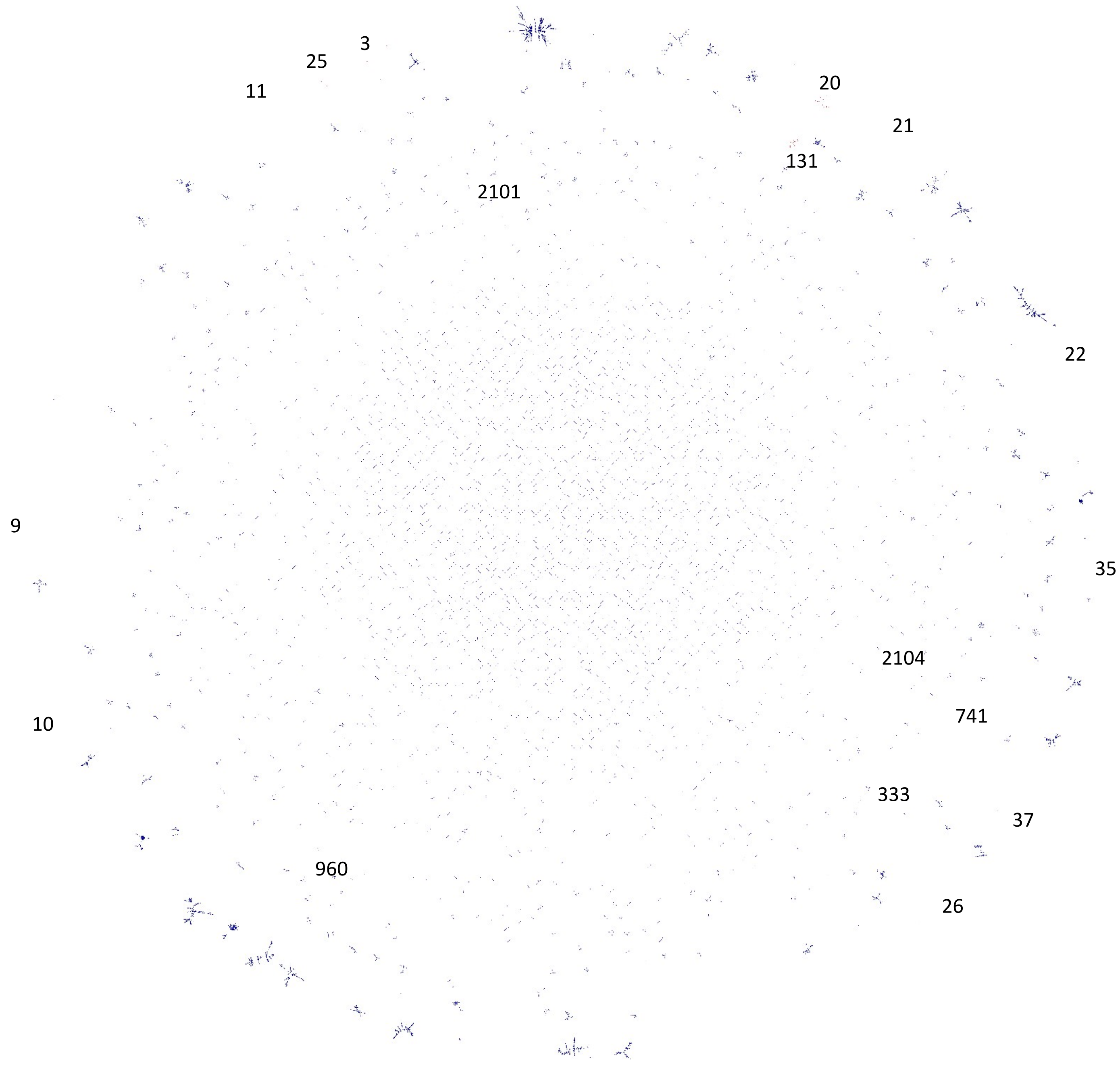

Supplementary Figure 2

b

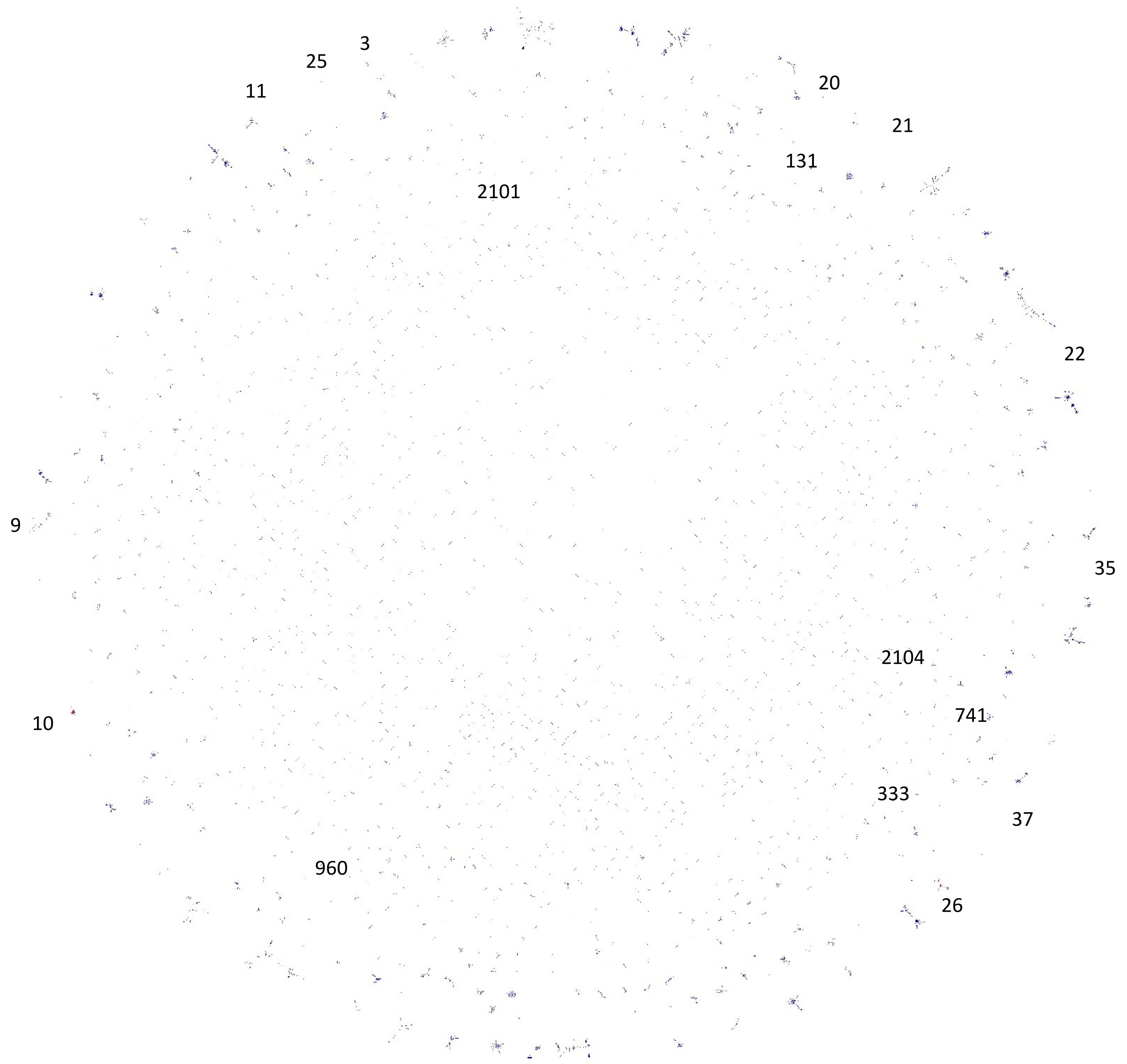

Supplementary Figure 2

c

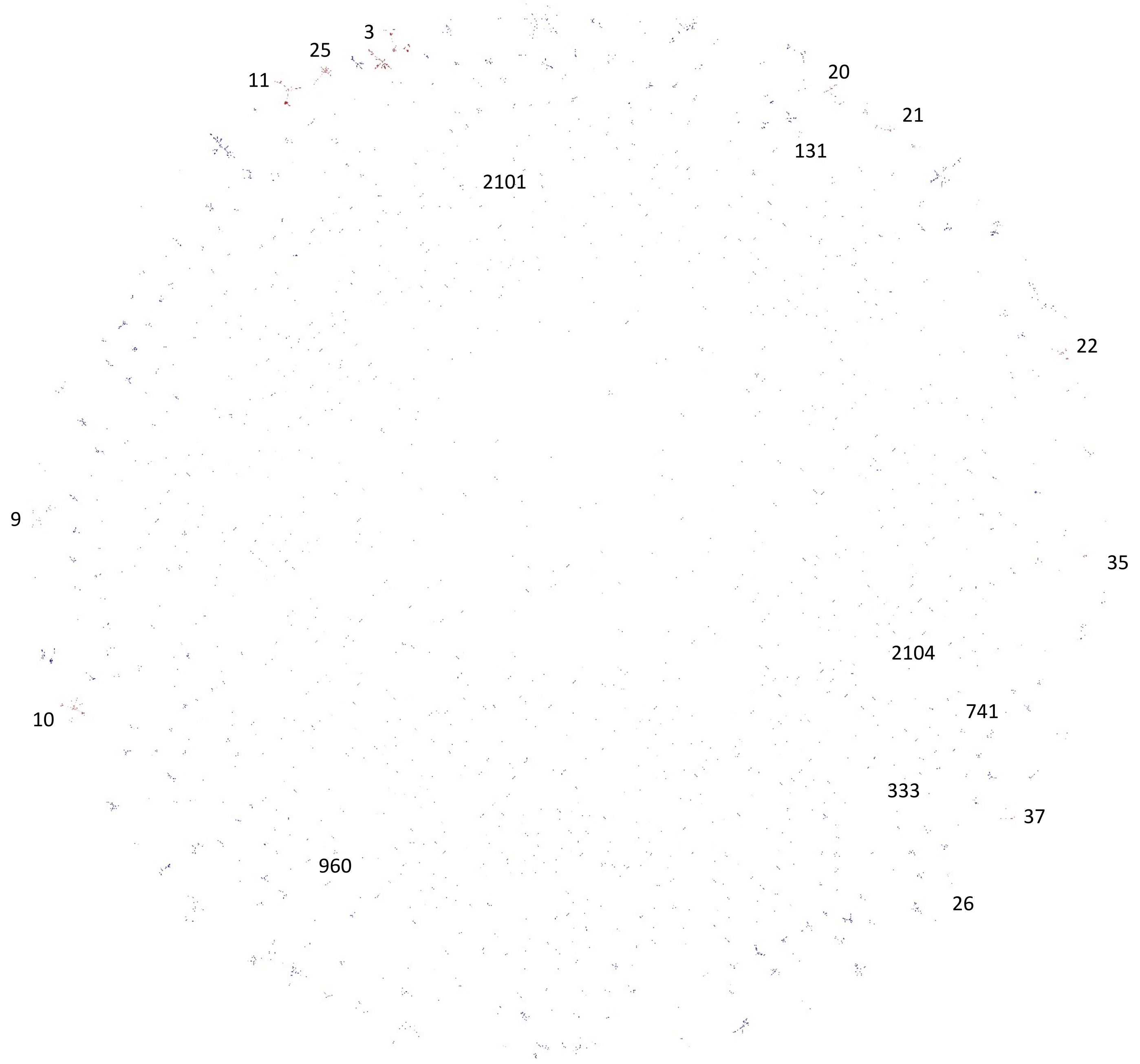

Supplementary Figure 2

d

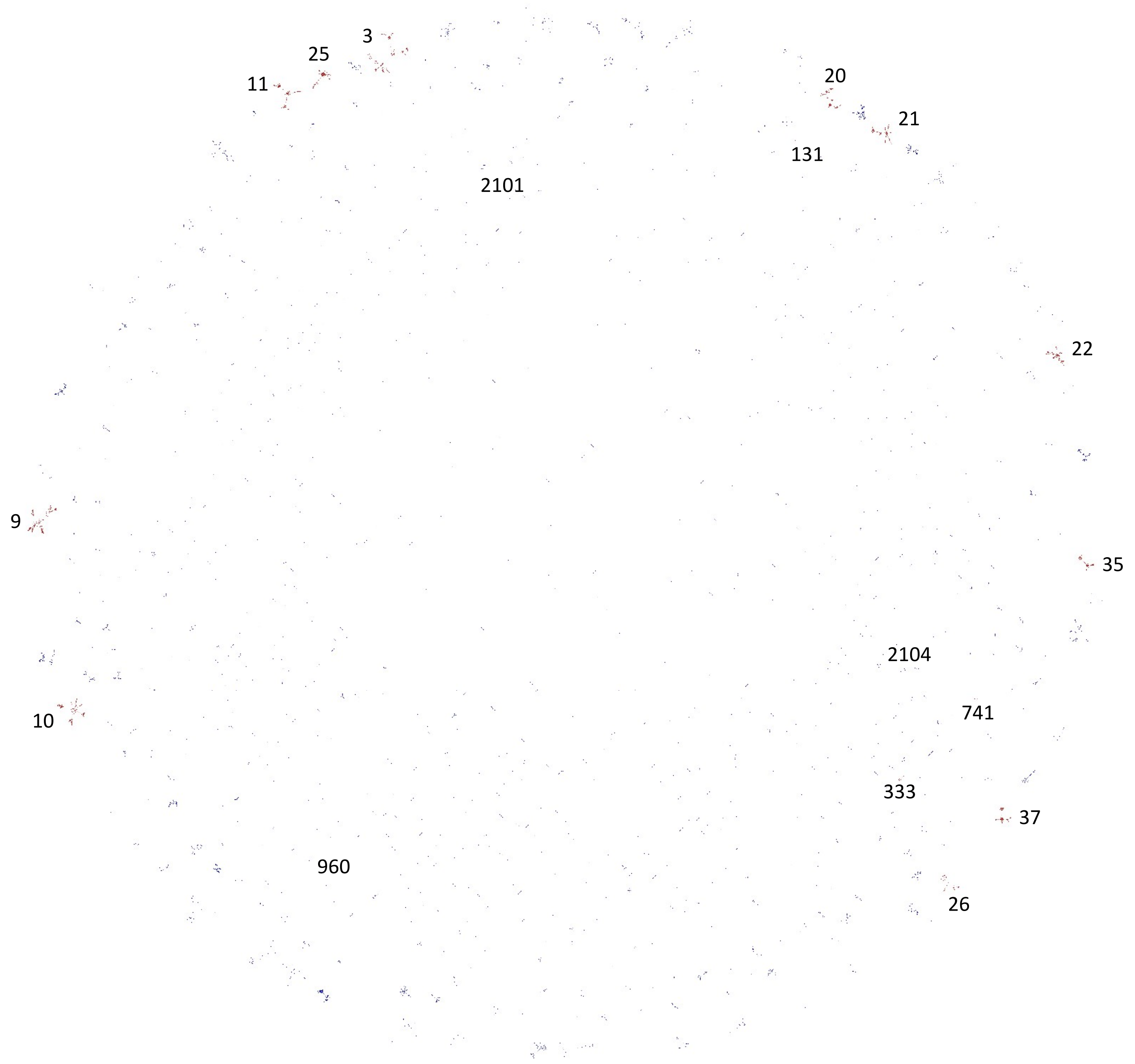

Supplementary Figure 2
e

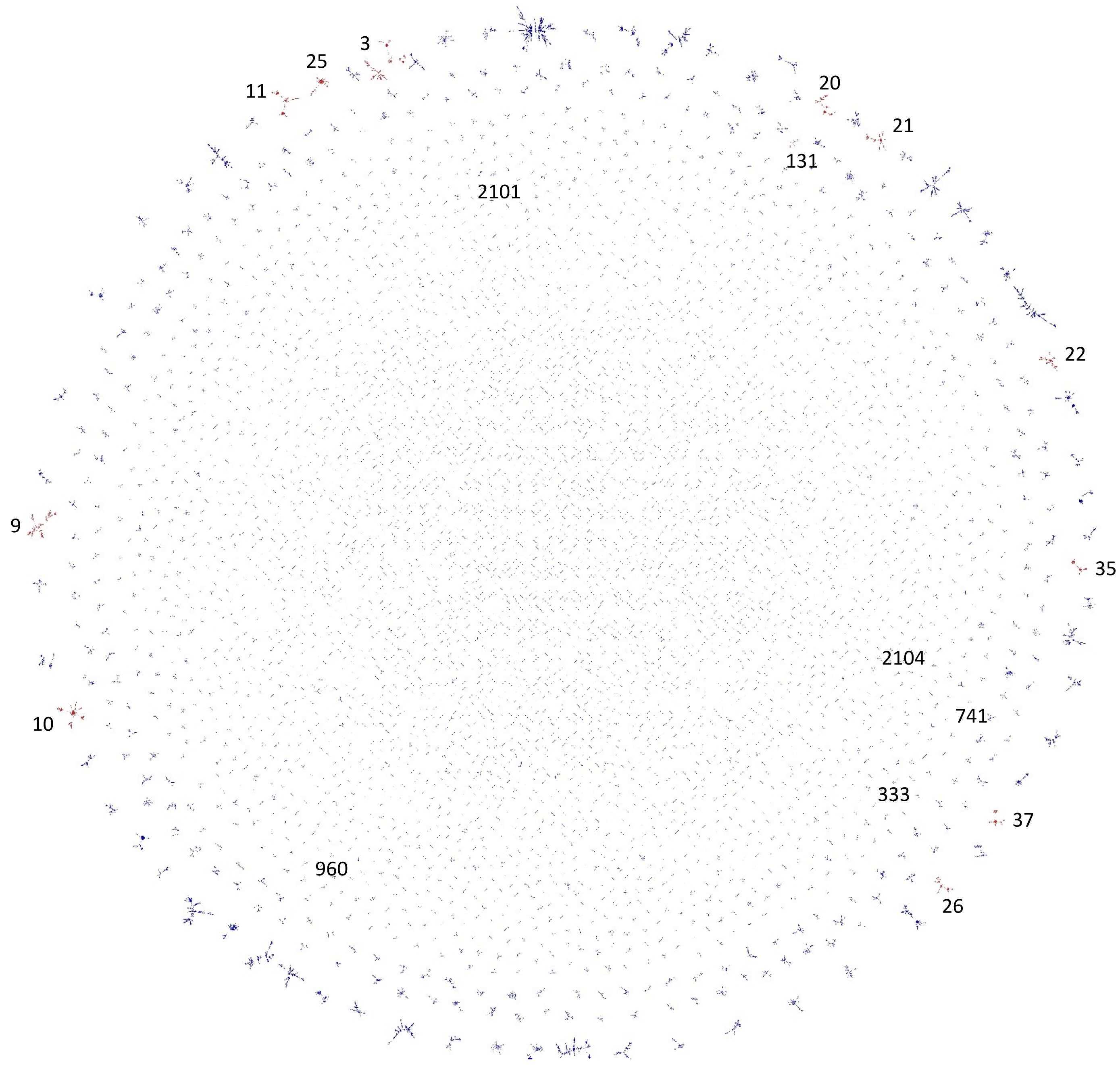

Supplementary figure 3

Figure S3**. Network image of V_H components in component 20 at weeks 0, 2, 4, and 5**

Network image of component 20 at week 0 **(a)**, 2 **(b)**, 4 **(c)**, and 5 **(d).** Vertices are labeled in descending order of clonal frequency a t week 5 and only vertices from one to 35 were marked numerical ly. The size of each vertex is proportional to its clonal frequency.

Supplementary Figure 3

a

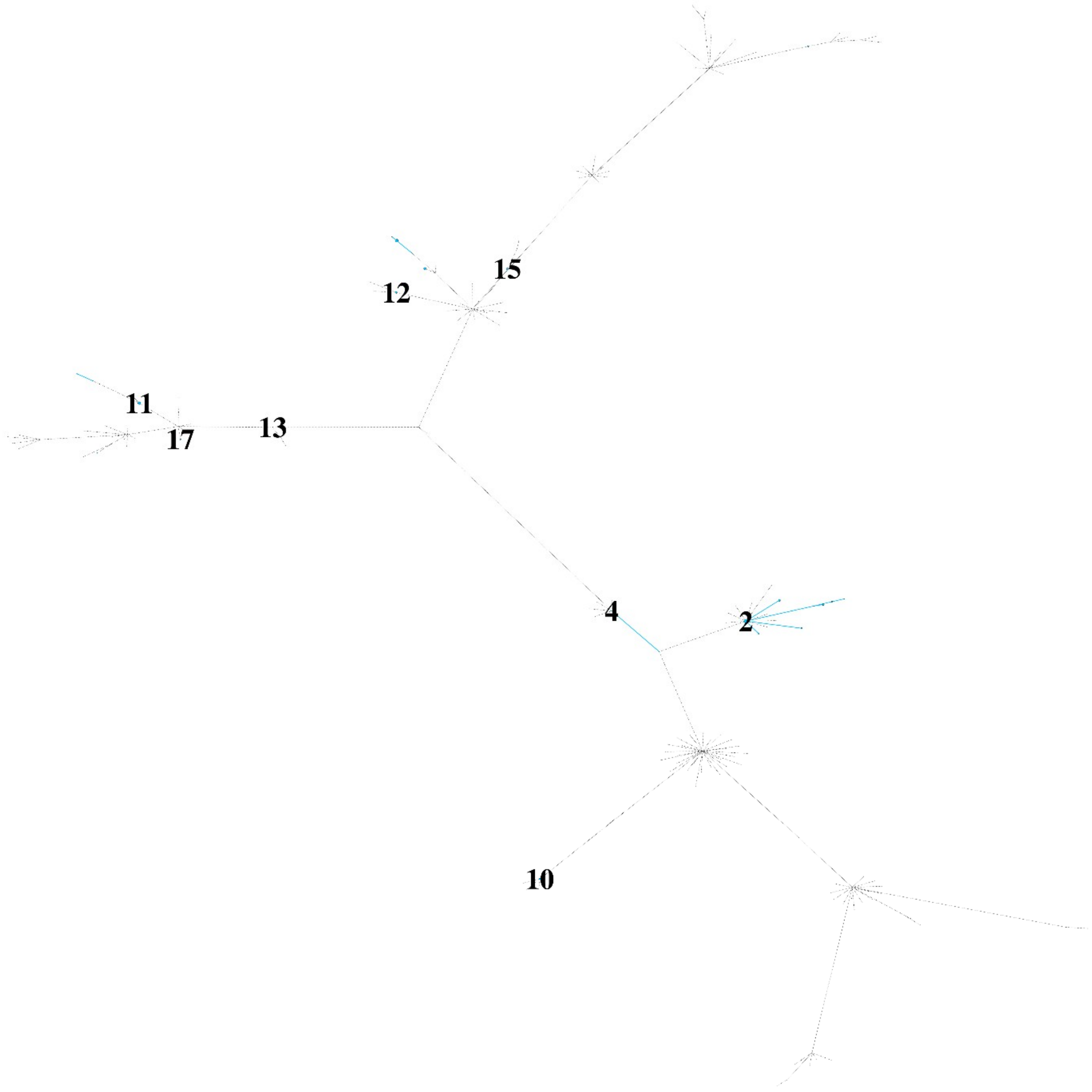

Supplementary Figure 3

b

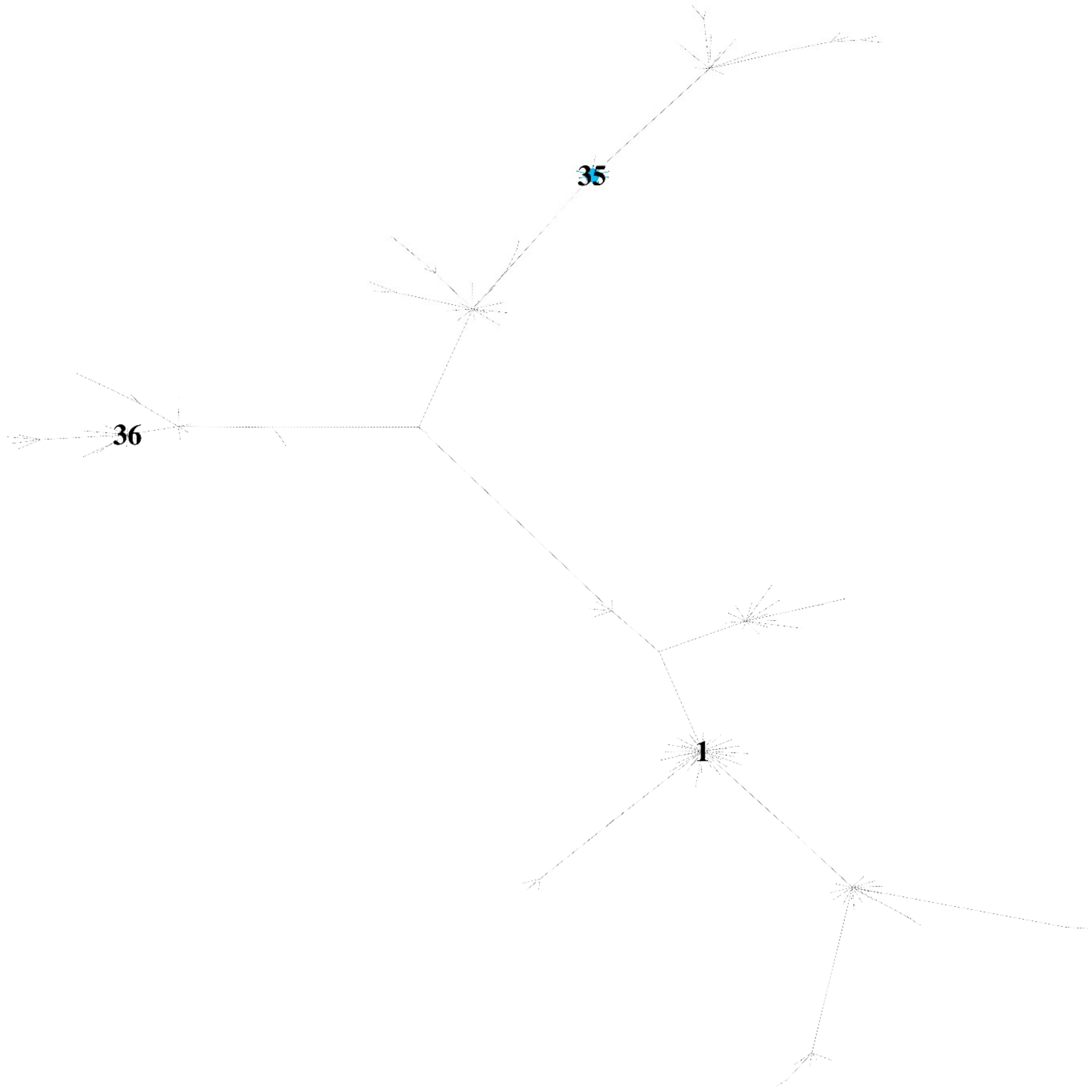

Supplementary Figure 3

c

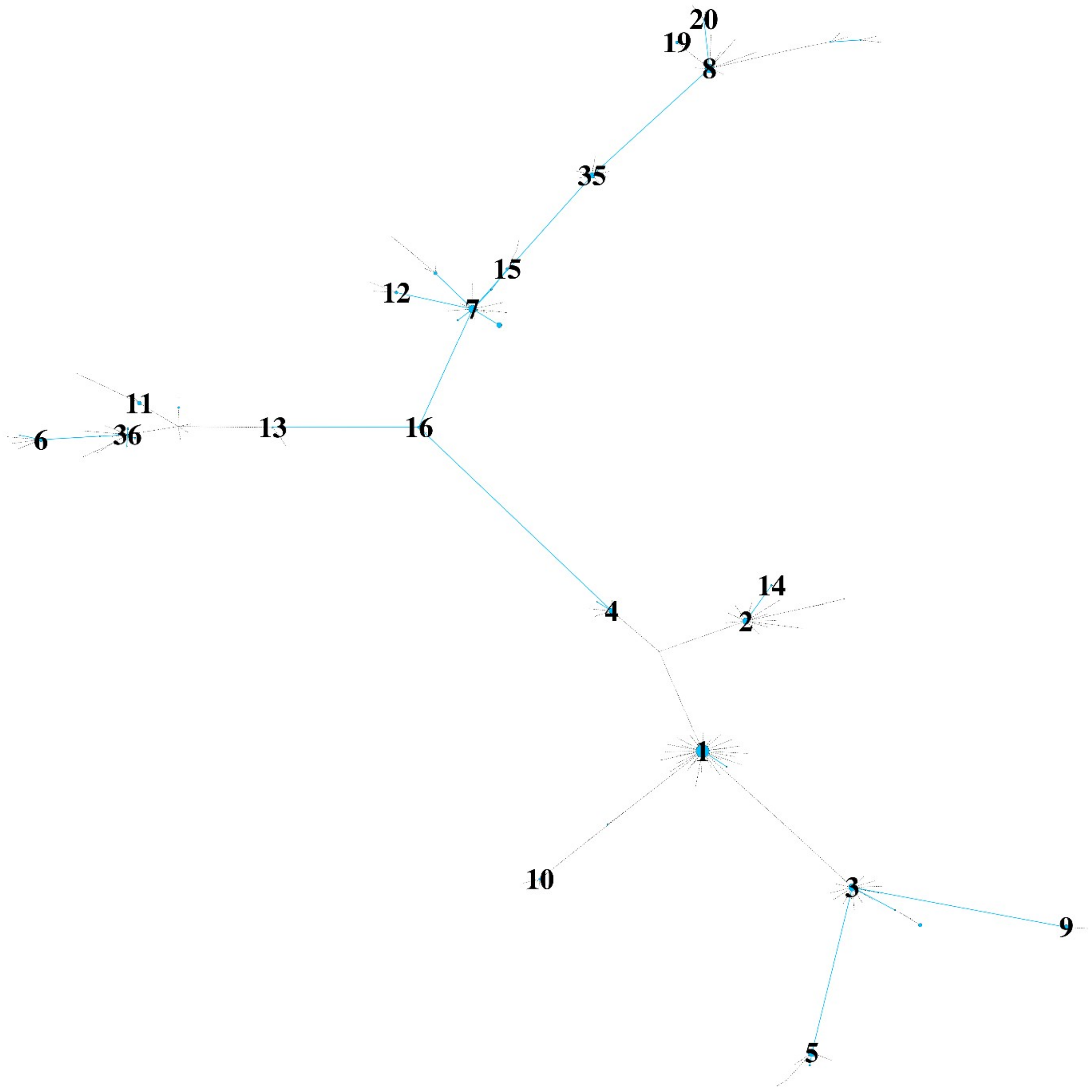

Supplementary Figure 3

d

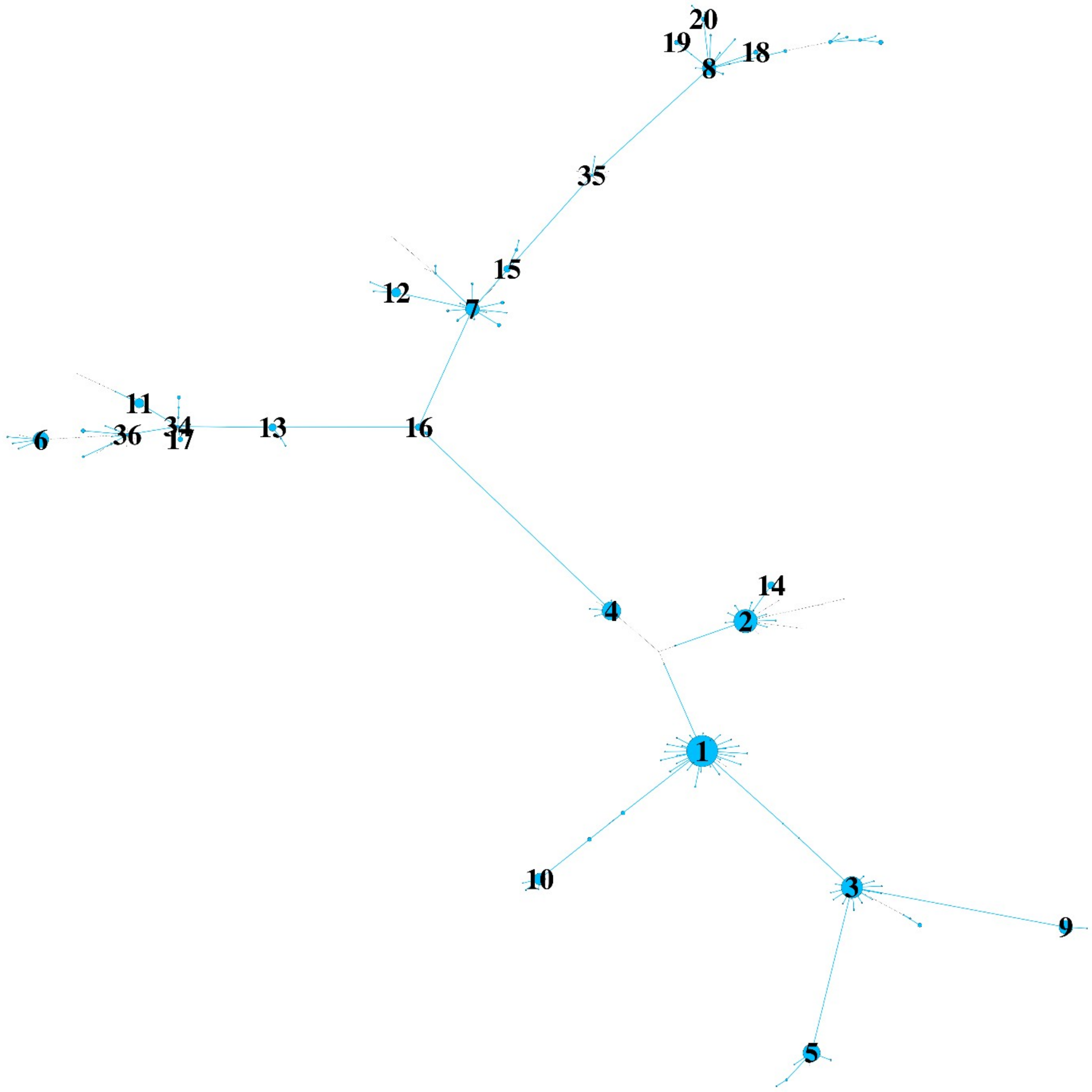

Supplement: Supplementary file 1 [file ijms-20-00417-s001.zip › supplementary/ijms-420679-supplementary figures.pdf]
