# Supplementary material for: Efficient Selection of Antibodies Reactive to Homologous Epitopes on Human and Mouse Hepatocyte Growth Factors by Next-Generation Sequencing-Based Analysis of the B Cell Repertoire
Source: Int J Mol Sci. 2019 Jan 18;20(2):417. doi: 10.3390/ijms20020417 (PMC6359367; doi:10.3390/ijms20020417)
Supplement: Supplementary file 1 [file ijms-20-00417-s001.zip › supplementary/ijms-420679-supplementary table.pdf]

Table S1. **Reactivity of phagemid clones to human HGF**

	Phagemid clones		
Components	Reactive to human HGF	Non-reactive to human HGF	Total
20	193	2	195
131	22	0	22
3	1	30	31
37	1	4	5
26	1	1	2
0	0	9	9
35	0	4	4
33	0	3	3
62	0	3	3
9	0	2	2
11	0	2	2
25	0	2	2
51	0	2	2
61	0	2	2
92	0	2	2
242	0	2	2
4	0	1	1
8	0	1	1
15	0	1	1
39	0	1	1
43	0	1	1
70	0	1	1
79	0	1	1
80	0	1	1
113	0	1	1
114	0	1	1
117	0	1	1
123	0	1	1
141	0	1	1
538	0	1	1
550	0	1	1
962	0	1	1
1360	0	1	1
2139	0	1	1
Total	218	88	306
